# Suicidal Ideation and Suicide-Attempt-Related Hospitalizations among People with Alzheimer’s Disease (AD) and AD-Related Dementias in the United States during 2016–2018

**DOI:** 10.3390/jcm11040943

**Published:** 2022-02-11

**Authors:** Golnoosh Alipour-Haris, Melissa J. Armstrong, Jennifer L. Sullivan, Uma Suryadevara, Masoud Rouhizadeh, Joshua D. Brown

**Affiliations:** 1 Center for Drug Evaluation & Safety, Department of Pharmaceutical Outcomes & Policy, College of Pharmacy, University of Florida, Gainesville, FL 32610, USA; g.alipourharis@ufl.edu (G.A.-H.); mrou@cop.ufl.edu (M.R.); 2 Norman Fixel Institute for Neurological Diseases, University of Florida, Gainesville, FL 32610, USA; melissa.armstrong@neurology.ufl.edu; 3 Department of Neurology, College of Medicine, University of Florida, Gainesville, FL 32610, USA; 4 Department of Health Services Policy and Practice, School of Public Health, Brown University, Providence, RI 02912, USA; jennifer.sullivan@va.gov; 5 Center for Innovation in Long Term Services and Supports, VA Providence Healthcare System, Providence, RI 02908, USA; 6 Department of Psychiatry, College of Medicine, University of Florida, Gainesville, FL 32610, USA; suryadevara@ufl.edu

**Keywords:** Alzheimer’s disease, ADRD, dementia, suicide attempt, suicidal ideation, suicidal behaviors

## Abstract

People living with Alzheimer’s disease (AD) and AD-related dementias (ADRDs) are at a higher risk of suicidal behaviors given intersecting risk factors. Previous studies generally only focused on AD, small clinical samples, or grouped all dementia subtypes together, limiting insights for other ADRD subtypes. The objective of this study was to generate evidence related to the relative burden of suicidal behaviors (suicidal ideation and suicide attempt) among people with AD and ADRDs. This retrospective cross-sectional study identified hospitalizations related to suicidal behaviors (suicidal ideation and suicide attempt) for patients with Alzheimer’s disease (AD) and AD-related dementias using ICD-10-CM codes from the Nationwide Readmissions Database (NRD). A logistic regression model was estimated to assess associations between AD/ADRD subtype and patient characteristics, and the risk for a suicidal-behavior-related hospitalization and modes of harm were reported. During 2016–2018, there were 12,538 hospitalizations related to suicidal behaviors for people with AD/ADRDs. The overall prevalence of suicidal-behavior-related hospitalizations was lowest for AD (0.8%) and highest for frontotemporal dementia (2.6%). Among hospitalizations for suicide attempts, the most common mode of harm was medications or drugs (89.2% of all attempts), followed by weapons (17.7%). We found that there was a difference in the frequency of suicidal-behavior-related hospitalizations among AD/ADRD hospitalized patients across dementia subtypes.

## 1. Introduction

There is a dichotomy of mental health in older adults. Many older adults report greater well-being and emotional capacity [1], yet suicide rates peak in older age groups [2,3]. Baby boomers have a higher suicide risk than prior generations, making suicide an important public health issue as this generation ages [4,5]. Presence of serious comorbid conditions, which are prevalent with increasing age, increase the risk of depression, anxiety, and suicidal behaviors [6].

People living with Alzheimer’s disease (AD) and AD-related dementias (ADRDs) are at a higher risk of suicidal behaviors given intersecting risk factors [7,8]. AD/ADRDs are associated with cognitive decline, personality changes, psychiatric illnesses, functional impairment, comorbid illnesses, dependence on formal and informal caregivers, and disease stigma [9,10]. Growing evidence suggests that the highest suicide risk is within the first year after AD/ADRD diagnosis [8]. A recent study in the Medicare population found a 54% increased rate of suicide in those with newly diagnosed AD/ADRD, compared to the general population. The highest risk was observed during the first 90 days after the initial AD/ADRD diagnosis [8].

Existing research generally only focuses on AD, small clinical samples, or groups all dementia subtypes together, limiting insights for other ADRD subtypes. Generalizing suicide risk across AD and ADRDs disregards key behavioral, psychiatric, and cognitive symptoms across dementia subtypes that may contribute to increased suicide risk [11,12]. For example, depression is twice as common in vascular dementia (VaD) than AD [13], frontotemporal dementia (FTD) is characterized with increased disinhibition and impulsivity [14], and Lewy body dementia (LBD) frequently presents with hallucinations and delusions [11,15]. In contrast, AD cognitive symptoms include loss of insight or awareness [16,17], which potentially reduces suicidal behaviors compared to other dementias as AD progresses.

The aforementioned Medicare study showed differences in suicidal-behavior risk in the first year after diagnosis between AD and ADRDs, including a lower risk for AD compared to VaD, LBD, and FTD [8]. A study in the U.S. Veteran population is the only known study to evaluate suicidal behaviors across AD and ADRD subtypes beyond the first year of diagnosis [12]. That study found the 2-year prevalence of suicidal behaviors (ideation, plan, or attempt) to be <1% in AD, but ~2.6% in vascular, 3.6% in frontotemporal, 1.7% in Lewy body, and 2.4% in mixed dementia [12]. Non-AD dementias also had a nearly 2-fold higher prevalence of mood, anxiety, and substance-use disorders than AD [12]. These results, suggest that ADRD subtype-specific evidence is needed in order to better understand and prevent suicide risk in the AD/ADRD population.

The objective of this study was to generate evidence related to the relative burden of suicidal behaviors (suicidal ideation and suicide attempt) among people with AD and ADRDs. We described the overall prevalence of suicidal behaviors among subtypes, associations between suicidal behaviors and AD/ADRD and other patient factors, and the modes of harm used in suicide attempts associated with a hospitalization.

## 2. Materials and Methods

This was a retrospective, cross-sectional study using the Nationwide Readmissions Database, developed by the Agency for Healthcare Research and Quality (https://www.hcup-us.ahrq.gov/nrdoverview.jsp; accessed on 1 February 2022). The Nationwide Readmissions Database includes all-payer hospital discharges from up to 30 states and consists of over 60% of all U.S. hospitalizations in community, public, academic, general acute care, and specialty hospitals. The data are publicly available and used under a data-use agreement with the data provider. The University of Florida Institutional Review Board approved this study as exempt (IRB201900471).

We identified hospitalizations for patients with AD and ADRDs using International Classification of Diseases, 10th Revision, Clinical Modification (ICD-10-CM) codes at any diagnosis position on the discharge record. ICD-10-CM codes included the following: AD (G30.xx), VaD (F01.5x), FTD (G31.0x), and LBD (G31.83). Mixed dementia was categorized if ≥2 dementia subtypes were present on the same record. Suicidal behaviors (suicidal ideation and suicide attempt) were categorized by an algorithm compiled by the Veteran’s Health Administration to track suicidal and other intentional or accidental self-harm behaviors [18]. Use of claims, and these coding algorithms, to identify suicide attempts had a high positive predictive value (PPV) [19].

Patient characteristics captured during hospitalization included age, sex, comorbid mental and physical conditions, and median household income (based on a patient’s ZIP code). Race was not available in our dataset. All comorbid conditions were categorized and captured using the Clinical Classification Software groupings and ICD-10-CM codes [20]. Mental conditions included depression, attention deficit hyperactivity disorder, anxiety, bipolar disorder, personality disorder, post-traumatic stress disorder, and psychotic disorders. Comorbid physical conditions included diabetes, hypertension, thyroid disease, substance use disorder, alcohol use disorder, asthma, chronic kidney disease, hyperlipidemia, atrial fibrillation, ischemic heart disease, rheumatoid or osteoarthritis, chronic pulmonary disease, congestive heart failure, liver disorders, and mobility disorders.

Overall prevalence of suicidal behaviors, and separately suicidal ideation and suicide attempts, were reported for AD, VaD, FTD, LBD, and mixed dementia-related hospitalizations. Patient characteristics were described stratified by dementia subtype. Due to a relatively small sample of suicide attempt hospitalizations (n = 1307), modes of harm were reported for the combined AD/ADRD group and were not separated by subtype. Modes of harm were described by specific substance or mode used (e.g., poisoning by benzodiazepines) and into broad categories (e.g., medications or drugs). Modes of harm were not mutually exclusive—an individual hospitalization could have more than one mode of harm reported.

A logistic regression model assessed associations between AD/ADRD subtype and patient characteristics and the risk for a suicidal-behavior-related hospitalization. Odds ratios (ORs) and 95% confidence intervals were reported. All analyses were conducted using SAS version 9.3 (Cary, NC, USA). We redacted all cell counts ≤11 in accordance with our data-use agreement.

## 3. Results

### 3.1. Frequency of Suicidal-Behavior-Related Hospitalizations in Dementia Subtypes

**Figure 1:**
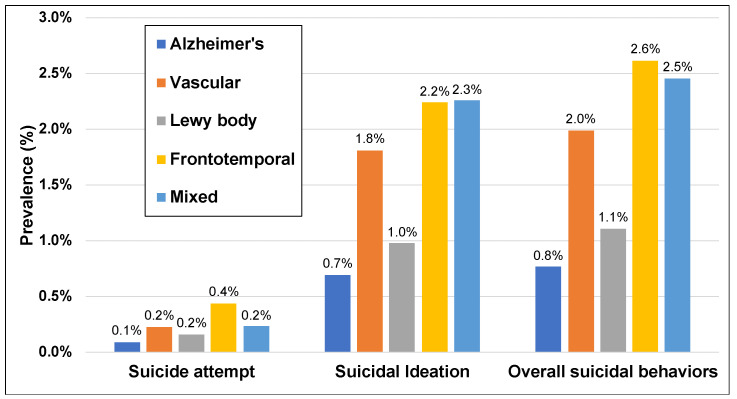
Prevalence of suicidal behaviors, including suicidal ideation and suicide attempt, in hospitalizations in people with Alzheimer’s disease (AD) and AD-related dementias. Data from the Nationwide Readmissions Database, 2016–2018, in the United States.

During 2016–2018, there were 12,538 hospitalizations related to suicidal behaviors for people with AD/ADRDs. Among hospitalizations for AD, the prevalence of suicidal behavior was 0.8% (5592) and, among all suicidal-behavior-related hospitalizations, 12.1% (578) were suicide attempts (Figure 1). VaD-related hospitalizations for suicidal behaviors were 2.0% (5035) of all hospitalizations, and 11.4% of these were for suicide attempts. Suicidal-behavior-related hospitalizations were 1.1% (578) of all LBD hospitalizations and 14.5% of these were for a suicide attempt. Mixed dementia-related hospitalizations for suicidal behaviors had an overall prevalence of 2.5% (963), and 9.4% of these were for suicide attempt.

### 3.2. Demographic and Clinical Characteristics

Patients with AD that were hospitalized for suicidal behavior had a mean patient age of 77 years, and 57% were female. The VaD cohort had a mean age of approximately 72 years and was 50% female, the LBD cohort had a mean age of 72 years and was 40% female, the FTD cohort had a mean age of 68 years and was 47% female, and the mixed dementia cohort had a mean age of 77 years and was 55% female. Depression was common and had similar prevalence across dementia subtypes (66–70%). Anxiety disorders were more common for those with FTD (44.3%) and mixed dementia (45.3%), compared to AD (40%). Bipolar disorder diagnoses were more common for ADRD subtypes and were highest for FTD (18.9%) and LBD (17.0%), compared to AD (12.7%). Personality disorders were very common for mixed dementias (23.6%), followed by FTD (7.3%), and there was a smaller prevalence with other dementia subtypes. Psychotic disorders were most common with LBD (33.9%) and FTD (31.4%), and AD had the lowest prevalence of 27.3%. Additional prevalence of comorbid conditions and other patient characteristics are shown in Table 1.

### 3.3. Associations between Patient Factors and Suicidal-Behavior-Related Hospitalizations

After adjusting for all measured characteristics, VaD, FTD, and mixed dementia, but not LBD, were associated with increased odds of suicidal-behavior-related hospitalization compared to AD (Table 2). For mixed dementia, the odds increased by 127% (OR = 2.27, 95% CI 2.02–2.54), and for VaD it increased by 85% (OR = 1.85, 95% CI 1.69–2.01). FTD was associated with a smaller increase of 35% (OR = 1.35, 95% CI 1.13–1.62).

Age and female sex were inversely associated with suicidal-behavior-related hospitalizations. For every 5 years, the odds of a suicidal-behavior-related hospitalization were 15% lower (OR = 0.85, 95% CI 0.85–0.87), and female sex reduced the odds by 23% (OR = 0.77, 95% CI 0.73–0.81). Mental health conditions were strongly associated with suicidal-behavior-related hospitalizations (Table 2).

### 3.4. Modes of Harm in Suicide Attempts

The most common broadly categorized mode of harm reported for 1307 suicide attempts was medications or drugs (1166; 89.2%) (Table 3). Weapons accounted for 17.7% of attempts and were primarily related to knives and sharp objects, rather than firearm use (data for firearms are not shown due to their small count) (Table 3). Asphyxiation accounted for 2.5% of attempts and included hanging or suffocation using other means (e.g., carbon monoxide poisoning). Mechanical harms made up 2.1% of all suicide attempts, which included jumping from high places and intentional vehicle- or transportation-related accidents. Alcohol (ethanol) or other substances were also reported in 1.5% of suicide attempts.

In more detailed coding descriptors (Table 4), benzodiazepines were the most reported medication for suicide attempts (233; 17.8%). Acetaminophen (7.8%) and opioids (7.0%) were also commonly reported medications in suicide attempts. Many suicide attempts had non-specific codes for the suicide attempt (17.1%) and self-harm (12.2%) without a mode of harm was identified.

## 4. Discussion

In this analysis of hospitalizations in a nationally representative sample of people with AD/ADRDs, the prevalence of suicidal behavior varied by dementia subtype. Overall prevalence of suicidal-behavior-related hospitalizations was lowest for AD (0.8%) and highest for FTD (2.6%). Among hospitalizations for suicide attempts, the most common mode of harm was medications or drugs (89.2% of all attempts), followed by weapons (17.7%).

Our results are comparable to previous findings in the U.S. Veteran population evaluating suicidal behaviors by AD/ADRD subtype. That study found the suicidal-behavior prevalence to be <1% in AD, but ~2.6% in vascular, 3.6% in frontotemporal, 1.7% in Lewy body, and 2.4% in mixed dementia [12]. The somewhat higher prevalence in their population may be related to several factors, most importantly differences in the population (armed forces Veterans vs. the general population) and capturing suicidal behaviors in other settings rather than just hospitalizations. Still, the relative differences between AD and ADRD subtypes was consistent.

Our overall findings for the association between AD and ADRD subtypes and suicidal-behavior-related hospitalizations were also similar to a study conducted in Medicare beneficiaries with AD/ADRDs [8]. In that study, ADRD subtypes were associated with higher suicidal-behavior risk and AD was associated with lower risk. Higher suicide risk in ADRD subtypes may be related to several factors, including condition-related factors. AD progression is characterized by loss of awareness, which may decrease perception of disease prognosis, and risk of suicide, as AD worsens. Comparatively, ADRD subtypes are associated with other cognitive and behavioral changes, such as depression in VaD, impulsivity in FTD, and psychotic symptoms in LBD [11,13,14,15]. Thus, as ADRD subtypes progress, suicide risk may increase with worsening psychiatric and behavioral symptoms. However this study did not evaluate the time course of suicidal behaviors in dementia subtypes, and other studies have not adequately investigated changing behaviors over time.

Age and female sex were associated with significant protective effects for suicidal-behavior risk in this study. The inverse association with older age may be related to cognitive functioning and awareness as dementia progresses, reducing the ability of the patient to present with suicidal ideation or carry out a suicide attempt [8]. In addition, as age increases, the issue of competing risks (i.e., other causes of death) likely decrease the likelihood of suicidal behaviors [21]. Female sex is generally associated with more suicide attempts but using less lethal means [22]. In the Medicare study, females with AD/ADRDs were 84% less likely to die from suicide compared to males and had 12% lower risk of any suicidal behaviors [8]. While we did not evaluate suicide mortality in this study, our findings of 23% lower suicidal behaviors for female sex were comparable to this prior study.

Factors leading to suicide attempt in older adults can be described using the Interpersonal Theory of Suicide. The ITS posits two, possibly modifiable, constructs, “thwarted belongingness” and “perceived burdensomeness” that cause suicidal desire. Thwarted belongingness results when the psychological “need to belong” is unmet. Perceived burdensomeness results when a person perceives they are a burden on others. Combined with “capacity” (overcoming the fear and pain of an attempt), these factors can lead to a suicide attempt [2]. These constructs result from other individual factors, such as social isolation, poor physical health, disability, and mental illness [2]. While we measured several related factors, including mental and physical conditions, other factors such as isolation, access to caregivers, and undiagnosed conditions not present in our database were likely missed or were not measurable in our dataset. Future work must evaluate these additional factors, especially with a goal to identify and intervene among those at highest risk. The current analysis adds to the evidence that AD and ADRD subtypes should be evaluated separately, given different disease progression and baseline risk for suicidal behaviors.

In this study, we found suicide-attempt-related hospitalizations in people with AD/ADRDs were mostly associated with poisoning (i.e., overdose is implied but not noted in ICD-10-CM descriptors) with medications or other drugs. This highlights the importance of patient and family counseling regarding access to high-risk medications, such as benzodiazepines, opioids, and acetaminophen. It also suggests potential practical strategies for limiting means, such as providing 30- rather than 90-day prescriptions for these medications. The mode of harm results must be interpreted with caution, however, given we only evaluated hospitalization records. Use of medications, drugs, and substances are less lethal than other means (i.e., weapons, asphyxiation, or mechanical), which may lead to life-sparing interventions or delayed death with hospitalization. In contrast, use of firearms, hanging, or falling events that are highly lethal may not report to a hospital for care. Additional data resources, such as death certificates, coroner reports, or police reports, are likely needed to discern causes of death for all suicide attempts in this, and other, clinical populations.

### Limitations

The Nationwide Readmission Database used has several strengths, including a nationally representative sample of all-payer hospitalizations. Prior studies have shown a higher risk of suicidal behaviors in those of White race compared to Black, Hispanic, and other races, as well as a positive association with Medicare low-income subsidies. Race was not included in our dataset, and we utilized a different measure of income aggregated by the median income of the patient’s ZIP code that was not patient specific. While suicidal behaviors have been associated with the first year after AD/ADRD diagnosis, this cross-sectional dataset did not allow for an assessment of diagnosis timing. The hypothesis that suicidal-behavior risk timing may differ between AD and ADRDs due to disease progression and symptomatology should be further explored. Lastly, suicidal behaviors captured using ICD-10-CM codes are likely to be highly underreported due to stigma and patient ability to convey intent. Using clinical notes and natural language processing in other non-AD/ADRD populations, researchers have found anywhere between 5–30 times more suicidal behaviors compared to diagnosis codes alone [23,24]. Applying similar approaches to the AD/ADRD population will improve our understanding of the epidemiology of suicidal behaviors in this population. However, regarding this study, suicidal-behavior codes do have high PPV, indicating a high proportion of true positives, which should be similar between subtypes [19]. Thus, the overall prevalence may be underestimated but the relative comparisons between dementia subtypes remain similar.

## 5. Conclusions

This study analyzed 12,538 hospitalizations related to suicidal behaviors for people with AD/ADRDs. The overall prevalence of suicidal-behavior-related hospitalizations was lowest for AD (0.8%) and highest for FTD (2.6%). Among hospitalizations for suicide attempts, the most common mode of harm was medications or drugs (89.2% of all attempts), followed by weapons (17.7%). We found that there was a difference in the frequency of suicidal-behavior-related hospitalizations among AD/ADRD hospitalized patients across dementia subtypes. Research and interventions targeted specifically to the unique behavioral and neuropsychiatric symptoms of dementia subtypes are needed to reduce suicidal behaviors in these populations.

## Figures and Tables

**Table 1:** Demographic and clinical characteristics of people with Alzheimer’s disease (AD) and AD-related dementias and a suicidal behavior-related hospitalization. Data from the Nationwide Readmissions Database, 2016–2018, in the United States.

	AD		VaD		LBD		FTD		Mixed	
ADRD population with suicidal behavior	N = 5592		N = 5035		N = 578		N = 370		N = 963	
**Age, mean (SD)**	77.0	0.3	71.7	0.4	71.9	0.9	68.3	1.0	77.4	0.6
**Female sex, n (%)**	3190	57.0%	2502	49.7%	230	39.8%	173	46.8%	525	54.5%
**Median household income, n (%)**										
Q1 (lowest income)	1671	29.9%	1594	31.7%	146	25.3%	82	22.2%	284	29.5%
Q2	1493	26.7%	1332	26.5%	146	25.3%	96	26.0%	256	26.6%
Q3	1274	22.8%	1087	21.6%	145	25.1%	102	27.6%	211	21.9%
Q4 (highest income)	1076	19.2%	947	18.8%	132	22.8%	82	22.2%	199	20.7%
**Mental health conditions, n (%)**										
Depression	3836	68.6%	3525	70.0%	387	67.0%	247	66.8%	680	70.6%
Anxiety	2238	40.0%	2107	41.8%	235	40.7%	164	44.3%	436	45.3%
Psychotic disorders	1526	27.3%	1392	27.6%	196	33.9%	116	31.4%	284	29.5%
Bipolar disorder	709	12.7%	772	15.3%	98	17.0%	70	18.9%	126	13.1%
Personality disorder	207	3.7%	247	4.9%	25	4.3%	27	7.3%	227	23.6%
PTSD	146	2.6%	210	4.2%	17	2.9%	20	5.4%	21	2.2%
ADHD	119	2.1%	145	2.9%	16	2.8%	17	4.6%	24	2.5%
**Other comorbidities, n (%)**										
Hypertension	4219	75.4%	3935	78.2%	418	72.3%	239	64.6%	783	81.3%
Hyperlipidemia	2727	48.8%	2571	51.1%	255	44.1%	152	41.1%	539	56.0%
Diabetes	1695	30.3%	1779	35.3%	158	27.3%	96	25.9%	286	29.7%
Chronic kidney disease	1591	28.5%	1628	32.3%	161	27.9%	76	20.5%	685	71.1%
Ischemic heart disease	1468	26.3%	1536	30.5%	137	23.7%	64	17.3%	297	30.8%
Thyroid disease	1333	23.8%	1072	21.3%	128	22.1%	87	23.5%	224	23.3%
Chronic pulmonary disease	1089	19.5%	1054	20.9%	71	12.3%	55	14.9%	160	16.6%
Rheumatoid or osteoarthritis	1070	19.1%	818	16.2%	57	9.9%	42	11.4%	180	18.7%
Atrial fibrillation	878	15.7%	882	17.5%	76	13.1%	40	10.8%	187	19.4%
Congestive heart failure	721	12.9%	788	15.7%	57	9.9%	32	8.6%	120	12.5%
Substance-use disorder	321	5.7%	558	11.1%	38	6.6%	35	9.5%	193	20.0%
Alcohol-use disorder	320	5.7%	480	9.5%	27	4.7%	24	6.5%	50	5.2%
Asthma	271	4.8%	250	5.0%	32	5.5%	21	5.7%	51	5.3%
Mobility Disorder	222	4.0%	462	9.2%	43	7.4%	13	3.5%	37	3.8%
Liver disorders	122	2.2%	161	3.2%	21	3.6%	**	**	66	6.9%

** Redacted due to low cell count (≤11). Abbreviations: ADHD—attention-deficit/hyperactivity disorder; PTSD—post-traumatic stress disorder.

**Table 2:** Association of patient demographic and clinical characteristics with suicidal behaviors among people with Alzheimer’s disease and related dementia hospitalizations.

Covariate	OR	95% CI
**Dementia subtype**		
Alzheimer’s disease	Reference	Reference
Vascular	1.85	1.69–2.01
Lewy body	0.97	0.85–1.10
Frontotemporal	1.35	1.13–1.62
Mixed	2.27	2.02–2.54
**Age (per 5 years)**	0.85	0.85–0.87
**Female sex**	0.77	0.73–0.81
**Median household income, n (%)**		
Q1 (lowest income)	Reference	
Q2	1.02	0.94–1.11
Q3	1.02	0.94–1.11
Q4 (highest income)	1.00	0.88–1.36
**Mental health conditions, n (%)**		
Depression	6.80	6.35–7.30
Psychotic disorders	2.77	2.56–3.00
Bipolar disorder	2.72	2.52–2.94
Personality disorder	2.47	2.12–2.90
ADHD	1.63	1.32–2.94
Anxiety	1.55	1.44–1.66
PTSD	1.43	1.23–1.67
**Other comorbidities, n (%)**		
Substance-use disorders	2.17	1.95–2.41
Alcohol-use disorder	1.78	1.61–1.98
Diabetes	1.03	0.98–1.08
Hypertension	1.02	0.96–1.08
Thyroid disease	1.06	0.99–1.12
Asthma	1.03	0.92–1.15
Hyperlipidemia	0.97	0.92–1.02
Atrial fibrillation	0.85	0.78–0.93
Ischemic heart disease	1.03	0.97–1.09
Rheumatoid or osteoarthritis	0.96	0.89–1.02
Chronic pulmonary disease	0.95	0.89–1.01
Liver disorders	0.70	0.60–0.82
Chronic kidney disease	0.65	0.62–0.69
Congestive heart failure	0.68	0.63–0.74
Mobility Disorder	0.52	0.47–0.58

Abbreviations: ADHD—attention-deficit/hyperactivity disorder; PTSD—post-traumatic stress disorder.

**Table 3:** Categories of methods of harm reported in hospitalizations for suicide attempts in patients with Alzheimer’s disease and related dementias, 2016–2018.

Method of Harm	N	%
Medications or drugs	1166	89.2%
Weapons (knives, firearms, etc.)	231	17.7%
Asphyxiation (hanging, carbon monoxide)	33	2.5%
Mechanical harms (falling, vehicles)	28	2.1%
Alcohol or other harmful substances	20	1.5%

Note: the denominator is the total number (1307) of suicide attempts. Methods of harm are not mutually exclusive, an individual record could have one or more reported.

**Table 4:** Detailed description of methods of harm reported on hospitalizations for suicide attempts in patients with Alzheimer’s disease and related dementias, 2016–2018.

Detailed Method of Harm Descriptions	N	%
Poisoning by benzodiazepines	233	17.8%
Suicide attempt (not specified)	224	17.1%
Self-harm by other specified means	160	12.2%
Poisoning by 4-aminophenol (acetaminophen)	102	7.8%
Poisoning by other opioids	91	7.0%
Intentional self-harm by knife	85	6.5%
Poisoning by other antiepileptic and sedative-hypnotic drugs	79	6.0%
Intentional self-harm by other sharp objects	60	4.6%
Intentional self-harm by unspecified sharp objects	56	4.3%
Poisoning by unspecified drugs, medicaments, and biological substances	54	4.1%
Poisoning by other antipsychotics and neuroleptics	52	4.0%
Poisoning by selective serotonin reuptake inhibitors	48	3.7%
Poisoning by selective serotonin and norepinephrine reuptake inhibitors	40	3.1%
Poisoning by beta-adrenoreceptor antagonists	39	3.0%
Poisoning by aspirin	31	2.4%
Poisoning by other drugs, medicaments, and biological substances	27	2.1%
Poisoning by other antihypertensive drugs	25	1.9%
Toxic effect of ethanol	23	1.8%
Poisoning by other synthetic narcotics	22	1.7%
Poisoning by antiallergic and antiemetic drugs	22	1.7%
Poisoning by tricyclic antidepressants	20	1.5%
Poisoning by propionic acid derivatives	19	1.5%
Poisoning by calcium-channel blockers	19	1.5%
Poisoning by angiotensin-converting-enzyme inhibitors	19	1.5%
Poisoning by other parasympathomimetics [cholinergics]	18	1.4%
Poisoning by other antidepressants	16	1.2%
Poisoning by tetracycline antidepressants	15	1.1%
Asphyxiation due to hanging	15	1.1%
Intentional self-harm by jumping from a high place	15	1.1%
Poisoning by antithyroid drugs	14	1.1%
Poisoning by unspecified synthetic narcotics	14	1.1%
Poisoning by antiparkinsonism drugs	13	1.0%
Poisoning by antihyperlipidemic and antiarteriosclerotic drugs	13	1.0%
Poisoning by thyroid hormones	12	0.9%
Other causes	273	20.9%

Note: the denominator is the total number (1307) of suicide attempts. Methods of harm are not mutually exclusive; an individual record could have one or more reported. “Other causes” reported due to requirement to redact low cell counts.

## Data Availability

Data are used under a data-use agreement that does not allow disclosure of source data. Programming codes and algorithms are available upon request from the corresponding author.
